# Oral nitrate and citrulline decrease blood pressure and increase vascular conductance in young adults: a potential therapy for heart failure

**DOI:** 10.1007/s00421-016-3418-7

**Published:** 2016-06-22

**Authors:** Paige Alsop, David Hauton

**Affiliations:** School of Food Science and Nutrition, Faculty of Maths and Physical Sciences, University of Leeds, Woodhouse Lane, Leeds, LS2 9JT UK

**Keywords:** Cardiac electrical activity, Nitrate, Citrulline, Vascular compliance

## Abstract

**Purpose:**

Both inorganic nitrate and citrulline are known to alter the arginine–nitric oxide–nitrate system to increase the bioavailability of nitric oxide with potential benefits in the treatment of heart failure. However, their effects on cardiac electrical activity, vascular compliance and peripheral conductance are less well understood. This study examined the effect of nitrate and citrulline on cardiac electrical activity and blood flow.

**Methods:**

Young adult subjects (*n* = 12) were recruited to investigate the effects of acute oral nitrate consumption (8 mg/kg) and chronic citrulline consumption (3 g/day) on cardiac electrical activity measured by ECG recording and blood pressure. Blood flow and vascular compliance were measured by IR-plethysmography at the thumb and the hallux.

**Results:**

Nitrate (*p* < 0.05) and citrulline (*p* < 0.01) consumption both decreased diastolic blood pressure but had no effect on either pulse pressure or rate-pressure product (NS for both). Citrulline also decreased systolic pressure (*p* < 0.01). Nitrate and citrulline both decreased vascular compliance (*p* < 0.05 for both) prior to isometric grip exercise, but this was increased for nitrate following exercise (NS). Citrulline decreased R–R interval 9 % (*p* < 0.05) at rest and increased heart rate (*p* < 0.05) in addition to significantly decreasing pulse transit duration (6 %; *p* < 0.05). QRS duration was also decreased by 5 % for citrulline (*p* < 0.05) with the reduction in R–R interval.

**Conclusion:**

Both nitrate and citrulline supplementation decreased vascular tone at rest but citrulline also altered sympathovagal balance to increase sympathetic tone. We suggest that both oral nitrate and citrulline may be suitable adjuvants for patients with heart failure to improve peripheral tissue oxygenation.

## Introduction

Heart failure (HF) is characterised by reductions in cardiac output and a decrease in the ability to deliver oxygen to peripheral tissues. This occurs as a consequence of multiple factors, including a decrease in cardiac contractility leading to a decrease in cardiac output. Perhaps more insidious amongst the consequences of HF is the increase in sympathetic tone which leads to an increase in heart rate (HR) initially and also increased vascular tone. As a result, ventricular filling is decreased hence contributing to a diminished cardiac output.

Therapies that increase ventricular filling without altering heart rate, therefore, have the potential to increase cardiac output without increasing cardiac oxygen demand through tachycardia. Acute exploitation of nitrate-releasing agents decreases heart rate, both systolic and diastolic blood pressures and, as a consequence, increases the left ventricular ejection fraction (Battock et al. 1976). Disadvantages to such therapies include poor potential to maintain systemic blood pressure. Amongst the therapies exploited include the organic nitrates (e.g. glycerol trinitrate-GTN) releasing nitric oxide in the systemic circulation, reducing left ventricular filling pressure and increasing cardiac index suggesting improvement in cardiac performance (Franciosa et al. 1978) through decreasing both preload and afterload (Kelly et al. 1990). Indeed, arteriolar and venular components of the cardiovascular system have differing sensitivities to GTN, with venous distension maximal at very low nitrate concentrations whilst arterial resistance shows lower sensitivities to GTN (Imhof et al. 1980). This holds the benefit for decreasing afterload whilst helping to preserve systemic arterial blood pressure. One shortcoming of such therapies is a gradual attenuation of the efficacy of organic nitrates (Leier et al. 1983).

More recently, inorganic nitrate found in certain fruit and vegetable juices has shown the potential to reduce blood pressure (Siervo et al. 2013) and for the improvement in cardiovascular parameters including vascular compliance (Lidder and Webb 2012). In addition, athletes have subsequently found ergogenic benefit from nitrate consumption with the potential to decrease systemic blood pressure and decrease oxygen consumption for fixed workloads (Larsen et al. 2010; Bailey et al. 2009; Vanhatalo et al. 2010). In healthy normotensive subjects, inorganic nitrate increased vascular compliance without altering flow-mediated dilatation (Bahra et al. 2012). Recent experiments also suggest that inorganic nitrate may also offer the potential to ameliorate disease; whilst consumption of nitrate-rich beetroot juice did not alter the exercise capacity of patients with COPD, oxygen consumption during exercise was decreased (Curtis et al. 2015). Furthermore, dietary nitrate improved endothelial function and decreased vascular stiffness in older adults (Rammos et al. 2014). All these effects have potential benefits in heart failure. Indeed, for patients with HF and preserved ejection fraction, nitrate supplementation increased exercise duration, total work and increased both peak oxygen extraction and cardiac output (Zamani et al. 2015).

Citrulline, the end product of arginine-mediated nitric oxide production, has shown the potential to increase plasma nitrate and NO availability (Schwedhelm et al. 2007; Morita et al. 2014). In addition, citrulline decreased blood pressure and does not demonstrate the desensitisation noted for GTN. Citrulline does not complex with haemoglobin and, therefore, decrease oxygen carriage, increases both plasma arginine and cGMP (Schwedhelm et al. 2007) and is free of side effects. In human subjects, citrulline decreased arterial stiffness in middle-aged men (Ochiai et al. 2012). Citrulline also attenuated the systolic pressure (SP) increase in response to the cold pressor test (Figueroa et al. 2010). More recently, citrulline has shown promise as an intervention for hypertension, reducing both blood pressure and cardiac augmentation index in obese pre- and hypertensive subjects (Figueroa et al. 2012). Together, these observations suggest that citrulline may also show benefit in human subjects for the improvement of oxygen delivery in heart failure. However, alterations to cardiac electrical activity and profound changes to blood pressure, heart rate or increases in tone of the sympathetic nervous system would preclude the use of citrulline.

We postulate that in healthy adults, both nitrate and citrulline supplementation will decrease blood pressure through peripheral vascular effects rather than decreasing heart rate. Nitrate and citrulline will also preserve cardiac electrical activity and will not alter sympathetic tone in response to acute increases in sympathetic nerve function, therefore, making citrulline a potential treatment for HF. We will examine the impact of nitrate and citrulline supplementation in young adults, measuring blood pressure and electrocardiogram characteristics. In addition, we will estimate peripheral blood flow parameters using infra-red (IR) plethysmography.

## Materials and methods

### Materials

Food grade salt petre (potassium nitrate) was purchased from Anglia Chemical Products (Ipswich, Suffolk, UK) and citrulline tablets were obtained from Source Naturals Inc. (Santa Cruz, CA, USA).

## Methods

### Ethical review

All experiments were approved by the Maths and Physical Sciences and Engineering joint Faculty Research Ethics Committee (MEEC), University of Leeds (Review Number: MEEC 14-028) and all studies complied with the Declaration of Helsinki. Young adults who were non-smokers were recruited to the experiments; criteria for exclusion included existing cardiac and kidney disease, diabetes, hypertension and Raynaud’s disease. All subjects gave their informed consent to participate in the experiments. Subjects attended the study facility on two separate occasions, separated by at least 1 week; all subjects were fasted overnight, well rested, had abstained from caffeine during the previous 12 h and had not undertaken vigorous exercise within the previous 24 h period.

### Experimental protocol

Anthropomorphic measurements including height and body mass were recorded. Body composition was estimated using bioimpedance analysis scales (Omron Healthcare Inc., Bannockburn, Illinois, USA). Maximum Voluntary Contraction (MVC) isometric grip (ISGE) strength was estimated using digital hand-grip using the non-dominant hand. Briefly, maximum grip strength was estimated following three maximum grip tests, a minimum of 30 secs apart. 30 % MVC was calculated from the mean of 3-maximal contractions. Oxygen saturation of haemoglobin (SaO_2_) was estimated using pulse oximeter (Anapulse, Ana Wiz Ltd, Surbiton, Surrey, UK) and data expressed as %-haemoglobin saturation.

All subjects were then instrumented to record peripheral pulse amplitude using IR plethysmograph attached to thumb and hallux (MLT-1020, AD Instruments, Oxford, UK). Blood pressure was measured using peripheral blood pressure cuff. ECG was recorded using 3-lead ECG recording equipment (Bio-Amp, AD Instruments, Oxford, UK) attached by adhesive electrodes to the inner surface of the dominant forearm, shoulder of the non-dominant arm and one ankle. All data were recorded via a datalogger (PowerLab 4/35, AD Instruments, Oxford, UK) to computer for further analysis. Subjects were seated in a relaxed position and spontaneously breathing.

**Fig. 1:**
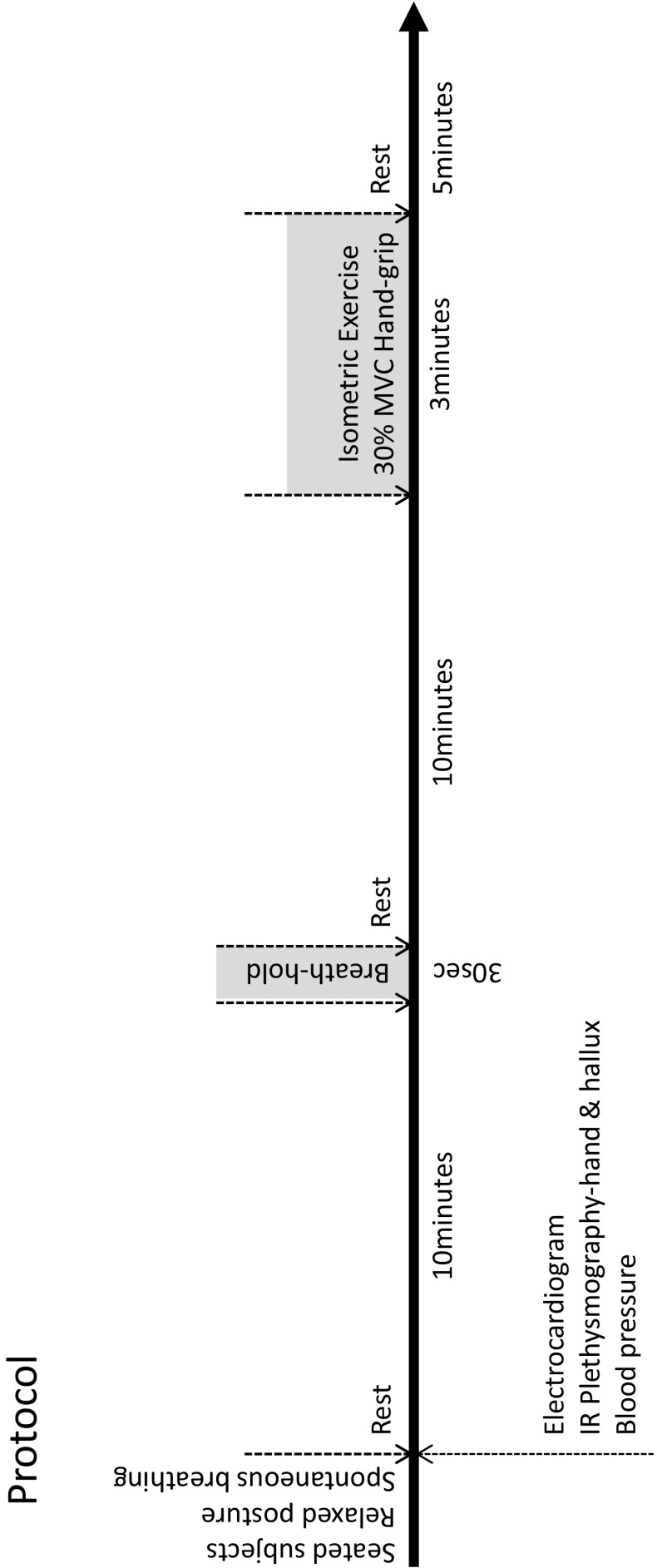
Experimental protocol timeline for human experiments

Blood flow and ECG were continuously recorded during 10 min rest period. Blood pressure was measured periodically (5 min). At 10 min, following a maximal inhalation a 30 s breath-hold was undertaken (Fig. 1), followed by a return to spontaneous breathing for a further 10 min. Subjects were then asked to sustain a grip test using the non-dominant hand estimated at 30 % MVC for 3 min. Blood flow and ECG measurements were continued throughout (Fig. 1). Following a return to baseline measurements, subjects were provided with sugar-free fruit cordial containing a nitrate supplement (8 mg/kg body mass) and remained within the study facility for a further 2 h prior to repeating the above test protocol. Following this second test, subjects were supplied with citrulline tablets (3 g/day) and asked to consume tablets for 7 days, in one single dose. At 7 days, subjects returned to the study facility to repeat the above test protocol (Fig. 1).

### Data analysis

Using 30 s averages, heart rate (HR) HR maximum and minimum and HR range were estimated. Systolic (SP) and diastolic pressures (DP) were recorded, with mean arterial pressure (MAP) calculated (MAP = [SP − DP]/3 + DP) and rate-pressure product (RPP) calculated as systolic pressure x heart rate. Pulse pressure (PP) was calculated as PP = SP − DP. Heart rate variability (HRV) was estimated from ECG using proprietary software (Chart 8.0, AD Instruments, Oxford, UK). Optimal settings for well-defined R waves were as follows: range 2 mV, high pass 0.3 Hz, low pass 50 Hz, sampling rate 1 kHz. The trace was used to calculate (beats min^−1^) and R–R intervals (ms), as well as the relative duration of the cardiac cycle components (sampled at 1 kHz). A minimum of 500 consecutive heart beats were examined. Peripheral pulse wave analysis in the time domain was undertaken using proprietary software (Chart 8.0, AD Instruments, Oxford), as previously detailed (Korpas et al. 2009; Allen and Murray 2002). To estimate the time delay between contraction of the ventricle and pulse delivery to the thumb or hallux pulse time was measured with regard to ECG. For 25 consecutive heart beats, pulse duration was measured as the duration between the R peak for an individual heart-beat and the pulse maximum for IR-plethysmograph recording for both thumb and Hallux. To estimate peripheral vascular compliance, the ratio of pulse amplitudes measured at the maximum and at the dicrotic notch was estimated for 25 consecutive heart beats at thumb (Klemsdal et al. 1996; Gunarathne et al. 2008).

Data are presented as mean ± SEM; statistical analysis was carried out using students ‘t’ test to examine the effects of nitrate or citrulline; analysis of variance (ANOVA) was used to quantify the effect of treatments with Bonferroni post hoc test to account for multiple comparisons.

## Results

### Anthropometry

12 subjects (mean age 22.8 ± 2.0 years; Table 1) enrolled for the experiment and all subjects completed both the nitrate and citrulline arms of the experiments. Nitrate supplementation decreased systolic pressure 3 %, but this was not significantly different from untreated subjects (NS; Table 1). By contrast, citrulline decreased SP 6 % (*p* < 0.01; Table 2). Diastolic blood pressure (DP) was reduced 6 % (*p* < 0.05; Table 2) by nitrate whereas citrulline decreased DP 14 % (*p* < 0.01; Table 2). By calculation, mean arterial pressure (MAP) was decreased 5 % by nitrate treatment (*p* < 0.05; Table 2), whereas citrulline reduced MAP 10 % (*p* < 0.01; Table 2). Rate-pressure product (RPP) was unaffected by either nitrate or citrulline treatment (NS for both; Table 2). Citrulline treatment decreased MVC by 7 % compared with untreated controls (*p* < 0.05; Table 2). Haemoglobin % oxygen saturation, measured by pulse oximetry, was unaffected by citrulline but was decreased 2 % by nitrate supplementation (*p* < 0.001; Table 2).

**Table 1:** Anthropometric characterisation of subjects

Parameter	Measurement
Age (years)	22.8 ± 2.0
Subjects (M/F)	12 (M = 4/F = 8)
Body mass (kg)	73.8 ± 3.0
Height (cm)	171 ± 2
Body mass index (kg/m^2^)	25.2 ± 0.7
Body fat mass (%)	31.0 ± 1.9
Muscle mass (%)	31.2 ± 1.5
Visceral fat (g)	5.8 ± 0.8

**Table 2:** Effects of nitrate and citrulline on cardiovascular parameters in human subjects

Measurement	Untreated	Nitrate (8 mg/kg)	Citrulline (3 g/day)
Systolic pressure (mmHg)	126.2 ± 3.3	122.1 ± 4.0	118.0 ± 3.7**
Diastolic pressure (mmHg)	78.0 ± 4.0	72.9 ± 2.7*	67.6 ± 2.8**
Mean arterial pressure (mmHg)	94.2 ± 3.3	89.3 ± 2.7*	84.4 ± 2.6**
Pulse pressure (mmHg)	48 ± 2	49 ± 4	50 ± 3
Heart rate (bpm)	76.9 ± 3.6	75.0 ± 3.0	78.0 ± 3.9
Rate-pressure product (mmHg/min)	9673 ± 473	9068 ± 249	9190 ± 467
Maximum voluntary contraction (kg)	37.5 ± 3.6	ND	35.0 ± 3.3*
Haemoglobin oxygen saturation [SaO_2_] (%)	98.1 ± 0.2	96.3 ± 0.4***	97.8 ± 0.2

Measurements of cardiovascular parameters for subjects at rest. Data represent mean ± SEM for *n* = 12 subjects

*ND* not determined

Statistical significance represented as different from untreated subjects: * *p* < 0.05, ** *p* < 0.01, *** *p* < 0.001

### Electrocardiogram

**Fig. 2:**
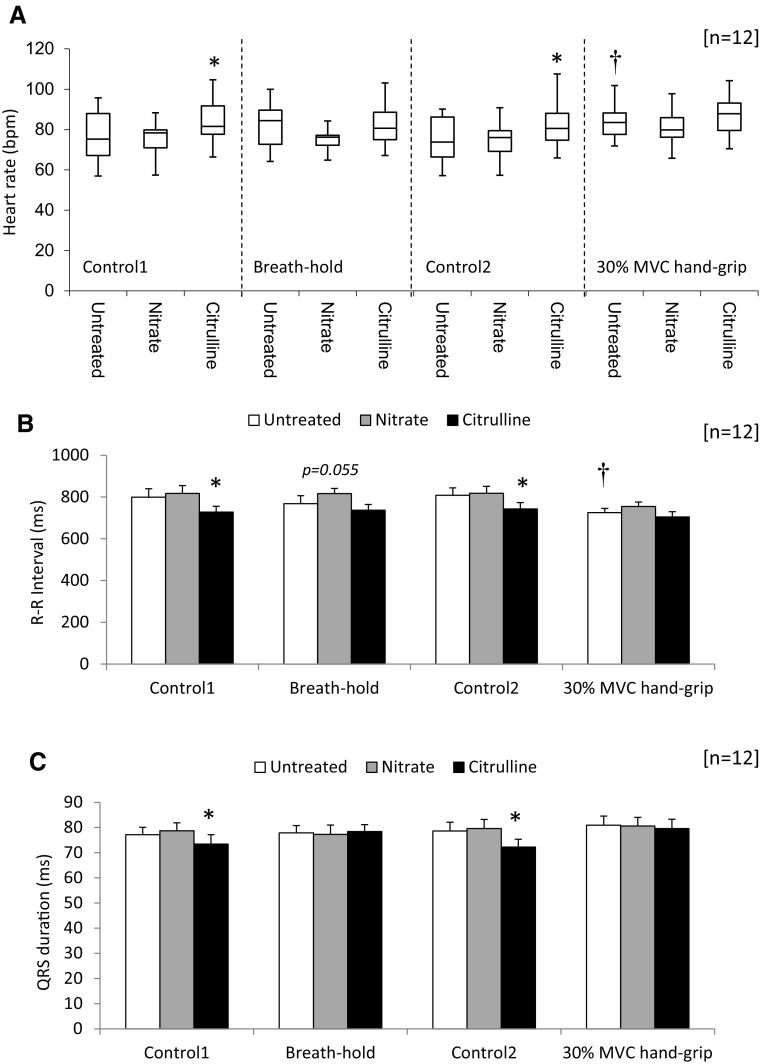
Cardiac electrical activity. Estimates of heart rate following nitrate and citrulline supplementation. Data represent maximum, 3rd quartile, median, 1st quartile and minimum HR (**a**). Estimates of cardiac R–R interval after nitrate or citrulline at rest (**b**). Measured QRS complex duration in subjects following nitrate and citrulline supplementation (**c**). For further details see methods. Data represent mean ± SEM (*n* = 12 subjects). Statistical significance indicated as: different from untreated control: *p < 0.05: effect of isometric grip exercise ^†^ *p* < 0.05

For untreated subjects, breath-hold increased HR 7 % (NS; Fig. 2a) whilst isometric grip exercise increased HR 11 % (*p* < 0.05; Fig. 2a). This was accompanied by corresponding decreases in R–R interval following ISGE (*p* < 0.05; Fig. 2b). Nitrate supplementation led to an 11 % decrease in HR during breath-hold (NS; Fig. 2a) accompanied by a corresponding increase in R–R interval (NS; Fig. 2b). Citrulline increased HR 9 % (*p* < 0.05; Fig. 2a) and decreased R–R interval significantly (*p* < 0.05; Fig. 2b) for subjects at rest. However, during both breath-hold and ISGE both HR (NS; Fig. 2a) and R–R interval (NS; Fig. 2b) were unchanged by citrulline supplementation.

Estimation of the domain-averaged durations for the cardiac events P, P-R, Q-T, Q-Tc, T, R-T, J-T and S-T was unchanged by either physiological intervention—breath-hold or ISGE—or by supplement—nitrate of citrulline (NS for all; data not shown). However, for subjects at rest, citrulline decreased QRS interval 5 % (*p* < 0.05; Fig. 2c), whilst QRS interval was unchanged following nitrate supplementation (NS; Fig. 2c).

### Pulse interval

**Fig. 3:**
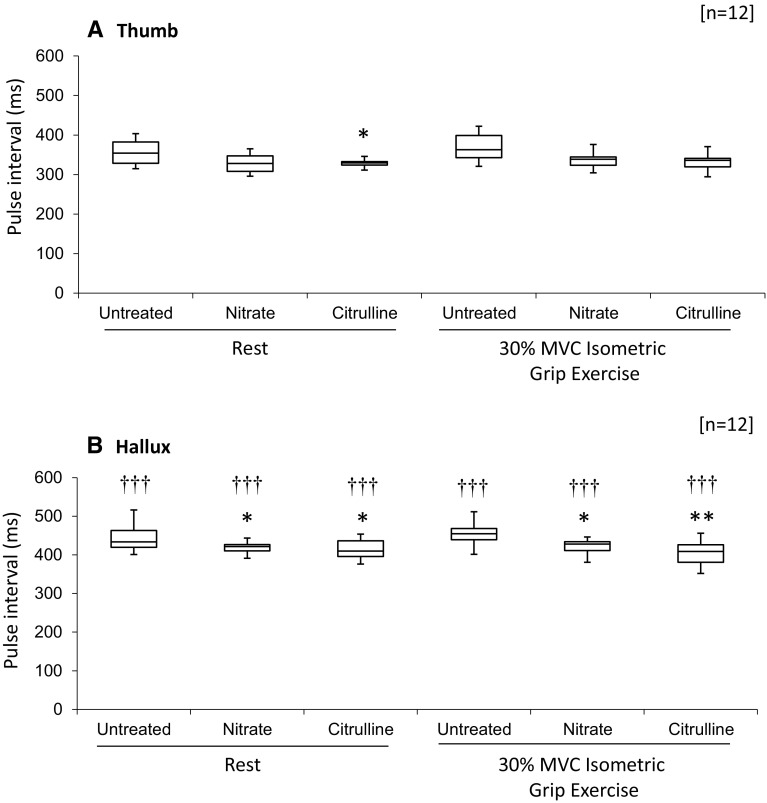
Pulse interval for peripheral blood flow at thumb (**a**) and Hallux (**b**). Pulse interval was calculated as duration between R–R interval and peak peripheral pulse. (**d**). For further details, see methods. Data represent maximum, 3rd quartile, median, 1st quartile and minimum HR (*n* = 12 subjects). Statistical significance indicated as: different from untreated control: *p < 0.05, **p < 0.01: different from pulse interval at the thumb ^†††^ *p* < 0.001

Pulse interval measured at the hallux was significantly greater than pulse interval measured at the thumb (*p* < 0.001; Fig. 3a, b). For subjects at rest, both nitrate and citrulline decreased pulse interval measured at the thumb 15 %, for nitrate treatment this did not reach statistical significance (NS; Fig. 3a), whereas for citrulline this was significantly different from untreated subjects (*p* < 0.05; Fig. 3a). For the hallux mean pulse interval was decreased 30 % by nitrate (*p* < 0.05; Fig. 3b) and decreased 35 % by citrulline (*p* < 0.05; Fig. 3b). Following ISGE, both nitrate and citrulline had no effect mean pulse interval measured at the thumb (NS for both; Fig. 3a) compared with untreated controls. However, mean pulse interval at the hallux was further decreased 35 % by nitrate (*p* < 0.05; Fig. 3b) and 55 % by citrulline (*p* < 0.01; Fig. 3b).

### Peripheral IR plethysmography

**Fig. 4:**
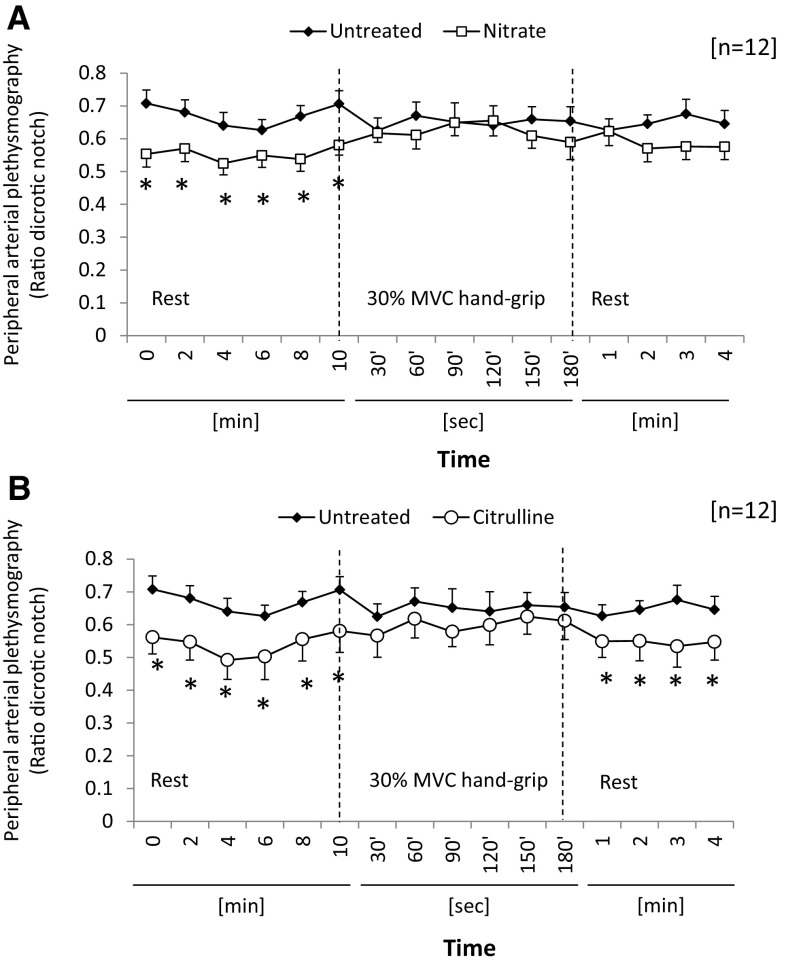
Pulse Amplitude Ratio measured by infra-red plethysmography. Effect of nitrate on pulse amplitude ratio (PAR) before and during isometric grip exercise (**a**). Effect of citrulline on PAR before and during isometric grip exercise (**b**). For further details, see methods. Data represent mean ± SEM (*n* = 12 subjects). Statistical significance indicated as: different from untreated control: *p < 0.05

Compliance of the peripheral vasculature, estimated from relative peak height of the dicrotic notch to the peak pulse amplitude (pulse amplitude ratio-PAR), was calculated from peripheral pulse signal. For untreated subjects, PAR was unchanged as a result of 30 % MVC-ISGE (NS; Fig. 4a). Following nitrate supplementation, PAR decreased 15 % (*p* < 0.05; Fig. 4a) at rest (Time *t* = 0 to *t* = 10 min). However, during ISGE this trend was lost (NS; Fig. 4a) and at completion of the exercise PAR showed a modest decrease to pre-exercise levels (NS; Fig. 4a). By contrast, citrulline supplementation significantly decreased PAR ~30 % during the rest period (*p* < 0.05; Fig. 4b); however, on the initiation of isometric grip exercise at 30 % ISGE PAR increased relative to untreated, such that it was not significantly different from the untreated controls (NS; Fig. 4b). During the subsequent recovery period, PAR declined following citrulline treatment and the significant difference from untreated subjects was restored (*p* < 0.05; Fig. 4b).

### Heart rate variability

**Fig. 5:**
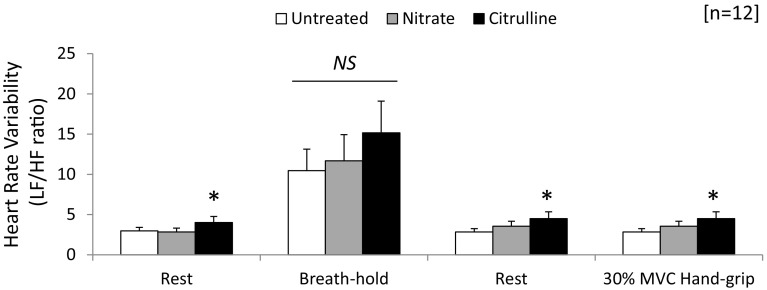
Estimate of sympathovagal balance following nitrate and citrulline supplementation. Data represent heart rate variability measurements made from ECG recordings for subjects at rest and during breath-hold and isometric grip exercise. For further details, see methods. Data represent mean ± SEM (*n* = 12 subjects). Statistical significance indicated as: different from untreated control: *p < 0.05

Heart rate variability (HRV) was estimated from ECG traces and data represented the balance of sympathetic outputs as the ratio between low frequency and high frequency outputs (Fig. 5). For subjects at rest, nitrate treatment had no effect on sympathovagal balance (NS; Fig. 5); however, citrulline treatment increased LF/HF by one-third, implying an increase in contribution from sympathetic nervous system (*p* < 0.05; Fig. 5). Irrespective of treatment, breath-hold increased sympathetic tone, measured as LF/HF ratio 4-fold (*p* < 0.001 for all; Fig. 5); however, neither nitrate nor citrulline had any additional effect on HRV estimates (NS; Fig. 5). ISGE at 30 % MVC did not increase measures of sympathovagal balance compared with the corresponding controls; however, the one-third increase noted for citrulline compared with untreated subjects was preserved (*p* < 0.05; Fig. 5).

### HR augmentation in response to exercise

**Fig. 6:**
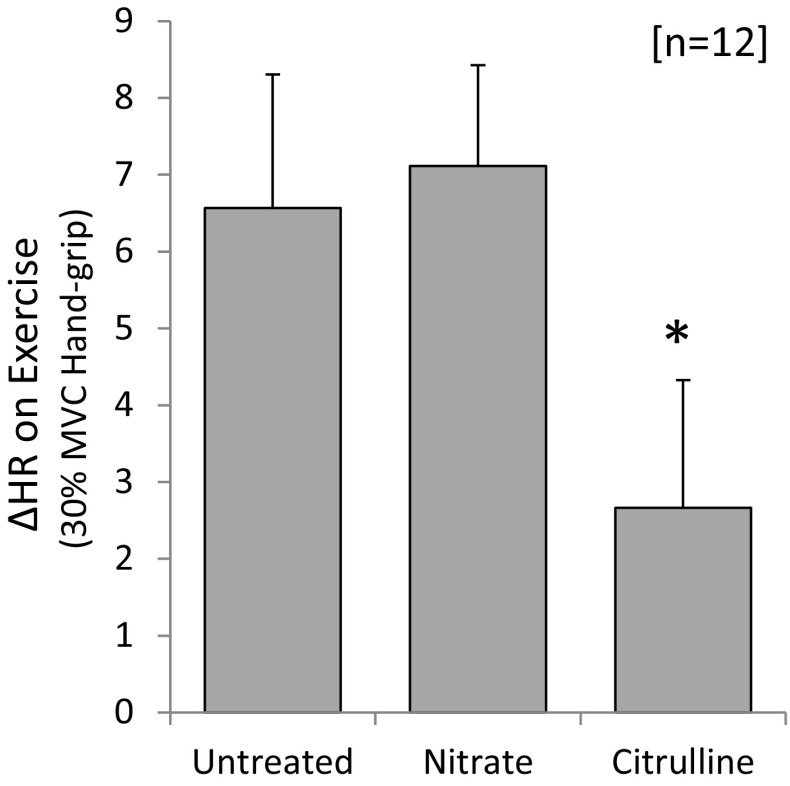
Augmentation in heart rate when transitioning from rest to isometric exercise. Data represent the effect of nitrate or citrulline on the increase in HR noted during 30 % MVC isometric grip test. For further details, see methods. Data represent mean ± SEM (*n* = 12 subjects). Statistical significance indicated as: different from untreated control: *p < 0.05

ISGE increased heart rate 6.6 ± 1.7 bpm for untreated subjects (Fig. 6). For nitrate-supplemented subjects, this increase was preserved, nitrate having no effect on HR augmentation during exercise. By contrast, the increase in HR during exercise was 2.7 ± 1.7 bpm for citrulline-treated subjects and hence the augmentation in response to exercise was approximately halved by citrulline (*p* < 0.05; Fig. 6).

## Discussion

Manipulation of the arginine–nitric oxide pathway has demonstrated the potential to improve cardiovascular function (Wu and Meininger 2000) and we confirm that either acute nitrate supplementation or chronic citrulline supplementation decreased mean arterial pressure and diastolic pressure, implying a decrease in total peripheral resistance (Cowley et al. 1973). Despite the apparent hypotension, cardiac rate-pressure product (RPP) was unchanged suggesting that cardiac work was unchanged. For citrulline, the fall in blood pressure was of sufficient magnitude to induce tachycardia to preserve RPP. In addition, apparent blood flow velocity, measured at the hand and foot, was also increased. This occurred despite a decrease in blood pressure, implying an increase in peripheral vascular conductance (Levitt et al. 2015) and suggesting improved tissue oxygenation. We, therefore, accept our hypothesis for cardiovascular improvements following nitrate and citrulline supplementation. Furthermore, assuming duplication of these effects in patients with HF we speculate that citrulline may potentially increase peripheral oxygen delivery.

We note a decrease in haemoglobin oxygen saturation (SaO_2_) following nitrate consumption which we propose is a consequence of methaemoglobin (mtHb) formation (Pluta et al. 2011). In contrast, studies exploiting beetroot juice, rich in nitrate, note no metHb (Kapil et al. 2015) and we postulate that this may relate to the use of KNO_3_ salt in cordial removing any potential matrix effects of food products and increasing rates of uptake. The onset and reversal of mtHb formation were previously noted to be rapid following the start and termination of nitrate infusion (Pluta et al. 2011), suggesting that rates of nitrate assimilation may contribute to methaemoglobinaemia. Despite the formation of mtHb, this reduction of SaO_2_ was asymptomatic (Pluta et al. 2011). Preservation of the pulse pressure despite decreases in diastolic pressure suggests that cardiac contractile performance, and hence stroke volume, is maintained and the decrease in MAP is a consequence of decreased peripheral resistance. Previous experiments demonstrated that both nitrate and citrulline supplementation induced acute vasodilatation in rodents (Chien et al. 2014), and increased nitric oxide production in vivo (Churchward-Venne et al. 2014; Schwedhelm et al. 2007). Dietary nitrate decreased DP in as little as 3 days (Larsen et al. 2006) whilst citrulline use in humans decreased both SP and DP blood pressure following 14-days supplementation (Sanchez-Gonzalez et al. 2012; Figueroa et al. 2010). Furthermore, supplementation of citrulline in human subjects increased plasma arginine (Moinard et al. 2008, 2015) and enhanced the bioavailability of nitric oxide (Morita et al. 2014). Together these observations imply that both nitrate and citrulline have the potential to decrease BP through direct release of NO or the mobilisation of substrate for NOS. Perhaps critical for subjects with compromised cardiac performance, neither nitrate nor citrulline altered RPP suggesting that cardiac work was preserved; therefore, despite a decrease in R–R interval and the modest tachycardia noted for citrulline, the overall burden in cardiac mechanical performance was unchanged. Given that ‘rate work’ consumes more oxygen than increasing developed pressure (Hauton et al. 2015), indicates that citrulline may yield a modest increase in cardiac oxygen consumption.

Surprisingly, citrulline modestly decreased maximum voluntary contraction for ISGE. This was unexpected and may result from the potential for excess nitric oxide to disrupt mitochondrial oxidative capacity (Bolanos et al. 1996; Clementi et al. 1998), but may also be a direct consequence of changes to blood pressure. Indeed, peak muscle blood flow is achieved at 30 % MVC-ISGE (Barnes 1980), and oxidative muscle performance is, in part, governed by blood pressure (Hobbs and McCloskey 1987).

Citrulline supplementation had only modest effects on cardiac electrical activity, including a small decrease in QRS interval duration for subjects at rest. Interestingly, when the apparent reduction in R–R interval is taken into account the QRS interval is preserved across all interventions, suggesting that our observations were a direct result of the modest tachycardia caused by citrulline. These observations were in direct contrast to earlier studies showing a decrease in QTc interval (Kameda et al. 2011) following acute consumption of citrulline. Whilst exploiting the same citrulline dose as used in our current experiment, these observations were made after acute consumption of citrulline (60 min) (Kameda et al. 2011) whilst our experiment demonstrates the effects of chronic citrulline consumption.

Estimates of blood velocity in the peripheral circulation measured from the pulse interval indicate that both exogenous nitrate and citrulline increase the flow velocity measured at both hand and foot. The origins of this increase in velocity may be different for both nitrate and citrulline. Nitrate, converted to nitric oxide, may increase the dilatation of vessels, decreasing the peripheral resistance. Evidence in support of this was the decreased diastolic pressure following nitrate consumption shown by ourselves and others (Levitt et al. 2015). The reduction in resistance with preserved cardiac performance, estimated as RPP, implies that flow and hence conductance have increased. Similar observations have been made following arginine supplementation in HF patients implicating the production of exogenous NO in the reduction of systemic vascular resistance (Bocchi et al. 2000). By contrast, the modest tachycardia caused by consumption of citrulline may also contribute to increasing flow velocity. Previous experiments in an elderly male population suggest that citrulline had no effect on blood flow in skeletal muscle (Churchward-Venne et al. 2014), although these authors postulate that the lack of effect for citrulline noted in their experiment may result from an inability to mobilise sufficient arginine to boost NO production. Moreover, impaired arginine transport has also been noted in HF (Kaye et al. 2000). Given that citrulline also has the potential to cause vasodilatation, secondary to an increase in plasma arginine (Moinard et al. 2008)—the substrate for nitric oxide synthase (NOS)—we cannot discriminate whether decreased vascular resistance or increased heart rate provides the greatest contribution to increases in flow velocity. Estimates of muscle oxygen utilisation measured using Near Infra-Red Spectroscopy (NIRS) have highlighted that both nitrate (Bailey et al. 2009) and citrulline (Bailey et al. 2015) may improve the efficiency of oxygen consumption during cycling exercise and, in consequence, may increase the time to exhaustion at fixed exercise loads. The origins of this improved efficiency (decreased oxygen extraction at fixed workloads) are unclear but may reflect a decrease in fractional oxygen extraction as a consequence of increased conductance, or increases in the efficiency of oxidative phosphorylation (Bailey et al. 2009, 2015).

The position of the dicrotic notch in the peripheral blood flow signal, estimated as PAR, reflected the general vascular tone in young adults (Klemsdal et al. 1996). Nitrate decreased the PAR at rest suggesting that a decrease in peripheral vascular tone (Bahra et al. 2012) was caused by nitrate or subsequent metabolites (Lund 1986). Previous experiments demonstrate that this effect is dependent upon nitric oxide or potential metabolites as arterial infusion of nitric oxide synthase inhibitors blunted arterial elasticity that was restored by arginine infusion (McVeigh et al. 2001). Furthermore, infusion of GTN increased arterial compliance (McVeigh et al. 2001).This would, in part, support the observations of potential increases in flow velocity as a consequence of decreased flow resistance. Interestingly, this effect was lost on the initiation of hand-grip exercise raising the PAR to levels noted for untreated subjects. On relaxation, the PAR failed to return to pre-exercise levels suggesting that generation of nitric oxide from nitrate was not restored to pre-test levels. The origins of this are unclear, but given that the half-time for NO is dependent upon pH, tissue oxygenation and the availability of nitrate (Kelm 1999) we cannot exclude the contribution from isometric exercise burden to NO breakdown. Indeed, superoxide radicals produced as a consequence of intense exercise may directly remove NO (Li and Forstermann 2000; Silvestro et al. 2002).

By contrast, citrulline, whilst decreasing vascular tone before isometric exercise, went on to restore reduced vascular tone directly after exercise. Previous experiments indicate that citrulline supplementation at similar doses to those used for our experiment decreased arterial stiffness in middle-age men (Ochiai et al. 2012), implying that a greater effect may be provided by decreased resistance (Sanchez-Gonzalez et al. 2012; Figueroa et al. 2010). This dichotomy may be a consequence of differences in the mechanism of action for both nitrate and citrulline. Namely, citrulline is an allosteric inhibitor of arginase enzyme, hence preventing the endothelial enzymic breakdown of arginine, the substrate for NOS, and raising the local arginine concentration (Berkowitz et al. 2006; Bailey et al. 2015) without increasing the plasma concentration of nitrate/nitrite (Hickner et al. 2006: Bailey et al. 2015). This may be less sensitive to prevailing effects of oxygen tension, superoxide production and pH than non-enzymic reduction of nitrate.

Examination of the heart rate variability (HRV) to quantify the balance between sympathetic and parasympathetic tone, indicated by the LF/HF ratio, suggests that nitrate had no effect on the sympathovagal balance in young adults. By contrast, citrulline increased the level of sympathetic tone modestly at both rest and during exercise. Such changes may be responsible for the modest tachycardia we noted for subjects consuming citrulline. Similar observations were noted for previous experiments exploiting the same dose of citrulline measured after only 60 min, but no direct measure of HR was made during these experiments (Kameda et al. 2011). Interestingly, the impact on sympathetic tone was modest as a challenge to strongly stimulate the sympathetic NS (a breath-hold) had no effect on the sympathovagal balance when compared with untreated subjects. The modest stimulus presented by isometric exercise confirmed the effect of citrulline on sympathetic tone and was corroborated by the lower augmentation in HR noted during exercise for citrulline-treated subjects.

### Experimental shortcomings

No estimates of dietary nitrate intake were made for the subjects. We have assumed that all subjects maintained an equivalent nitrate intake throughout the experiment. Variability in our data for nitrate-treated subjects may be improved by estimating plasma nitrate or citrulline concentrations or restricting nitrate consumption. These may be useful additions in future experiments. Previous experiments estimate that the pharmacokinetics of citrulline indicate that the plasma half-life for citrulline may be 0.9 h (Moinard et al. 2008) compared with 5–8 h for dietary nitrate (Tannenbaum 1979) suggesting that the time delay between citrulline consumption and repeat experimentation may be critical. However, supplementation of older adults with citrulline elevated plasma arginine concentrations for up to 6 h (Moinard et al. 2015; Schwedhelm et al. 2007) suggesting little effect on plasma arginine levels during our sampling window. Our experiment takes no account of gender differences or stage of the menstrual cycle for the female subjects. Interestingly, previous experiments suggest that different stages of the menstrual cycle had no effect on cardiac QT interval or autonomic tone (Burke et al. 1997) and indicate that gender differences in cardiac electrical activity are not a consequence of oestrogen (Burke et al. 1997). More recently, Minson et al. (2000) noted no effect of menstrual cycle stage on cardiovagal baroreflex sensitivity or vascular resistance. Together these imply that the changes we note are not likely to reflect difference in oestrogen status for female subjects.

## Conclusions

We demonstrate that both nitrate and citrulline decrease systemic blood pressure and increase vascular conductance, with the potential to increase peripheral tissue oxygenation. These effects occur without altering cardiac electrical activity suggesting that they may be safe adjuvants to cardiac therapies for patients with heart failure. Citrulline modestly alters sympathovagal balance to increase sympathetic tone but this does not alter the vascular response to either citrulline or nitrate. Future experiments aim to investigate the effects of both nitrate and citrulline on peripheral tissue oxygenation and exercise capacity in HF and determine whether citrulline may perpetuate the increases in sympathetic tone noted in HF.

## Abbreviations

BP: Blood pressure
DP: Diastolic pressure
GTN: Glyceryl trinitrate
HF: Heart failure
HR: Heart rate
HRV: Heart rate variability
ISGE: Isometric grip exercise
LF/HF: Low frequency/high frequency ratio
MAP: Mean arterial pressure
MVC: Maximum voluntary contraction
NO: Nitric oxide
NOS: Nitric oxide synthase
PP: Pulse pressure
RPP: Rate-pressure product
SaO_2_: Oxygen saturation of haemoglobin (%)
SP: Systolic pressure
Δ: Change
